# Statistical Uncertainty in Paleoclimate Proxy Reconstructions

**DOI:** 10.1029/2021GL092773

**Published:** 2021-08-01

**Authors:** H. L. O. McClelland, I. Halevy, D. A. Wolf‐Gladrow, D. Evans, A. S. Bradley

**Affiliations:** ^1^ School of Geography, Earth and Atmospheric Sciences University of Melbourne Parkville VIC Australia; ^2^ Department of Earth & Planetary Sciences Washington University in St. Louis St Louis MO USA; ^3^ Department of Earth & Planetary Sciences Weizmann Institute of Science Rehovot Israel; ^4^ Alfred‐Wegener‐Institut Helmholtz Zentrum für Polar‐ und Meeresforschung Bremerhaven Germany; ^5^ Institute of Geosciences Goethe University Frankfurt Frankfurt Germany

**Keywords:** paleoclimate, paleoceanography, proxies, inverse prediction, calibration, confidence intervals

## Abstract

A quantitative analysis of any environment older than the instrumental record relies on proxies. Uncertainties associated with proxy reconstructions are often underestimated, which can lead to artificial conflict between different proxies, and between data and models. In this paper, using ordinary least squares linear regression as a common example, we describe a simple, robust and generalizable method for quantifying uncertainty in proxy reconstructions. We highlight the primary controls on the magnitude of uncertainty, and compare this simple estimate to equivalent estimates from Bayesian, nonparametric and fiducial statistical frameworks. We discuss when it may be possible to reduce uncertainties, and conclude that the unexplained variance in the calibration must always feature in the uncertainty in the reconstruction. This directs future research toward explaining as much of the variance in the calibration data as possible. We also advocate for a “data‐forward” approach, that clearly decouples the presentation of proxy data from plausible environmental inferences.

## Key Points

- Statistical uncertainties in proxy reconstructions are dominated by unexplained variance in the calibration
- Proxy data should be presented as explicitly distinct from environmental interpretations
- Future efforts should prioritize collection of multiple explanatory variables for calibrations, and true replicate paleoanalyses

## 1 Introduction

Ancient environments cannot be observed directly, so paleo‐environmental reconstructions rely on indirect proxy approaches. A proxy is a characteristic of a material that can be measured, and is known to correlate with some aspect of the material's environment of formation. A paleo‐environmental proxy reconstruction is complete only with a quantitative statement of its uncertainty (Taylor & Kuyatt, 1994). A number of proxy‐specific statistical models now exist for a handful of mature proxies, however, statistical uncertainty in proxy reconstructions remains widely underestimated, particularly for new proxies. Our aim is to explore the factors that control the magnitude of uncertainty in reconstructions, and to provide a simple, robust and generalizable approach that can be used to estimate the uncertainty associated with any paleo‐environmental proxy reconstruction.

The first step in proxy reconstruction is to constrain a relationship between the proxy variable to be measured (*P*) and the environmental variable to be inferred (*E*) with a calibration data set consisting of paired values. For all proxies, the value of *P* depends on the value of *E*, so this relationship must be defined with a forward model, where *E* is the independent variable, and *P* is the dependent variable. Calibration data can be generated in laboratory experiments by varying *E* independently from all other influential variables (e.g., Bemis et al., 1998; Kluge et al., 2015), or by analyzing natural environmental samples, taking advantage of natural temporal or spatial variation in *E* (e.g., Anand et al., 2003; Dekens et al., 2002; Gray et al., 2018; Kim et al., 2008). The second step is to estimate the value of *E* that led to the value of *P*, which involves “inverting” the calibration model. As *P* is always a function of multiple environmental variables in addition to *E*, there is always residual variance in the calibration data, which reflects the complex variability of the natural world. It is this residual variance that usually dominates the uncertainty in proxy reconstructions, yet this component of uncertainty is often ignored.

In our analysis, we focus on the most common type of linear relationship between *E* and *P* where the three prerequisites for ordinary least squares (OLS) linear regression are satisfied. Despite its simplicity, OLS linear regression highlights the major problems common to all proxy approaches, and enables us to explore the controls on the magnitude of uncertainty in a reconstruction. A number of widely used proxies are based on linear calibrations, and any nonlinear, multivariate and more complex models can also be coerced into a linear form by algebraic manipulation or by plotting the model‐predicted values against the observations.

## 2 Calibration

A calibration data set consists of paired values of *E* and *P* (i.e., ($E_{i}$, $P_{i}$), i = 1, …, *n*). In the calibration context, *E* is considered to be an independent non‐stochastic variable. The value of *P* is influenced by *E*, in addition to other, often unknown, factors that are treated in the current approach as noise. *P* is, therefore, a dependent stochastic variable. By convention, the independent variable (*E*) is plotted on the *x*‐axis, and the dependent variable (*P*) is plotted on the *y*‐axis.

In our example we consider a calibration data set that conforms to the assumptions of the Gauss‐Markov theorem. This requires that the noise: (a) has zero mean; (b) has a single variance (is homoscedastic); (c) comprises uncorrelated values. The intercept ($\beta_{0}$) and the slope ($\beta_{1}$) of the straight line model are estimated by OLS linear regression, where the line minimizes the sum of squared differences between model *P* values and observations of *P*. This is a “*P*‐on‐E” OLS linear regression. The data pairs ($E_{i}$, $P_{i}$; *i* = 1, …, *n*) are then related by the forward model:

(1) $$P_{i}=\beta_{1}E_{i}+\beta_{0}+\epsilon_{i},$$

where ϵ is a noise term. We here assume that the noise is normal ($\epsilon_{i}∼N\left(0,\;\sigma^{2}\right)$), but this is not a requirement for OLS regression. When one of the prerequisites for Equation 1 is not satisfied, an alternative method of regression is required. Nonlinear and multivariate models will not be discussed here, nor will univariate linear models that account for uncertainties in both variables, but these are thoroughly reviewed elsewhere (e.g., Smith, 2009).

**Figure 1:**
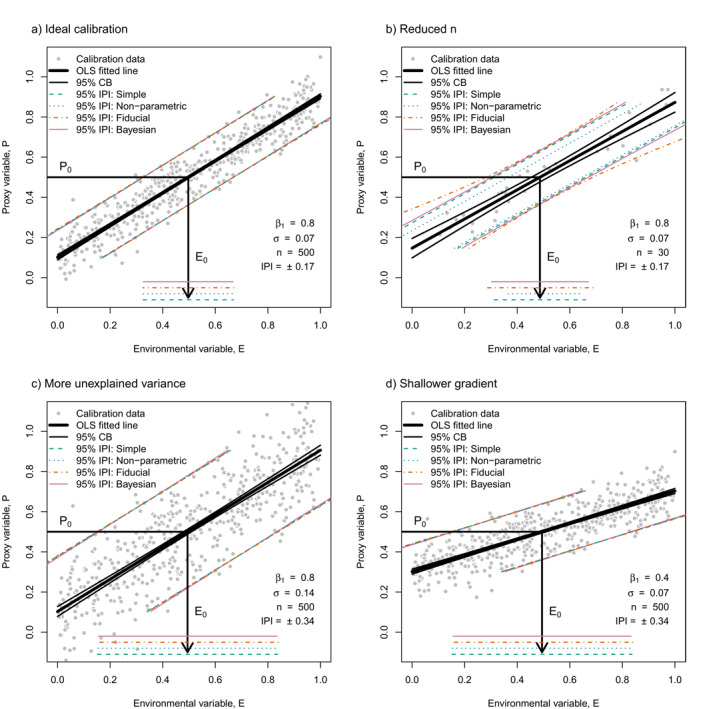
*Proxy calibrations with associated inverse prediction intervals*. Linear regression analyses on an artificial data set of an environmental variable (*E*), and a measurable proxy variable (*P*). The artificial data set was generated with Gaussian noise in the *P* axis alone. The regression lines were calculated with a *P*‐on‐*E* ordinary least squares (OLS) linear regression. The confidence bands (CBs) of the regressions represent the 95% uncertainty in the model parameters intercept and slope. 95% inverse prediction intervals (IPIs) in *E* are given by four different approaches: 1. Simple IPI (this study: Section 3); 2. Nonparametric IPI (Text S1); 3. Fiducial IPI. 4. Bayesian IPI (strictly, credible interval; Text S2). The “true” parameters $\beta_{1}$ (slope) and σ (noise), and the sample size, *n,* are prescribed for each condition. All approaches yield similar values of uncertainty in the estimated value of $E_{0}$, for a given value of $P_{0}$ of 0.5, represented by horizontal lines at the bottom of each plot. (a) An ideal calibration consisting of 500 data points. (b) Calibration consisting of 30 data points picked randomly from (a). Note the line is less well constrained but the IPI is similar to (a). (c) Calibration with all parameters as (a), except double the noise. Note the line is well constrained but the IPI is double that of (a). (d) Calibration with all parameters as (a), except a slope half as steep. Note the line is well constrained but the IPI is double that of (a).

This regression model is defined by three parameters: $\beta_{0}$, $\beta_{1}$ and σ. Estimates of these parameters are respectively denoted: ${\hat{\beta }}_{0}$, ${\hat{\beta }}_{1}$ and $\hat{\sigma }$. Classically, uncertainties in estimates of $\beta_{0}$ and $\beta_{1}$ are quantified and illustrated by construction of confidence bands (CBs; Figure 1) valid over all *E* values (hyperbolic CB [Scheffé, 1959]) or over restricted intervals (straight lines [Gafarian, 1964]). CBs are constructed in such a way that, under repeated experiments, the long‐run average of CBs that cover the true line is the nominal probability, say, 95% (see Casella & Berger, 2002 for a detailed discussion). In the Bayesian approach, all model parameters are estimated by a posterior distribution that integrates information from the data and the model assumptions. Uncertainties in the parameter values decrease with increasing n, typically according to $1/\sqrt{n}$. Parameter uncertainties can, therefore, be conceptualized as analogous to standard errors, where larger calibration data sets result in greater confidence in the model. However, while more calibration data improves estimates of the intercept, slope and magnitude of the noise, the same is not true for the uncertainty associated with inverse prediction.

## 3 Inverse Prediction

### 3.1 Inversion of the Forward Model

The goal of a paleo‐environmental proxy is to estimate *E* from *P*. For a typical linear relationship where *E* is the independent variable; this is called *inverse prediction*. The classical estimator for *E* (${\hat{E}}_{0}$; e.g., Osborne, 1991), can be straightforwardly derived from Equation 1 as follows:

(2) $${\hat{E}}_{0}=\frac{P_{0}-{\hat{\beta }}_{0}}{{\hat{\beta }}_{1}},$$

where $P_{0}$ is a single new value of the proxy variable, and where ${\hat{\beta }}_{0}$ and ${\hat{\beta }}_{1}$ are respectively the estimates of $\beta_{0}$ and $\beta_{1}$ constrained from OLS regression. The question is: what is the feasible range of values of $E_{0}$ that could have given rise to the observed value $P_{0}$? Here we call this the inverse prediction interval (IPI).

Quantifying the IPI is not trivial (e.g., Eisenhart, 1939; Hoadley, 1970, and see Osborne, 1991 for an excellent review). Frequentist inference, which is the primary statistical framework with which most scientists are familiar, is based on an assumption that the characteristics of the system have fixed values that can be constrained with the addition of data. As *E* is an independent variable, the uncertainty distribution parallel to this axis is not defined, so this statistical framework is not appropriate for estimating the IPI. This is the so‐called “calibration problem” (Shukla, 1972).

There are, however, a number of alternative approaches to frequentist inference that can be used to estimate the uncertainty in the value of $E_{0}$ for a given $P_{0}$. First, in the Bayesian statistical approach, the uncertainty distribution in $E_{0}$ can be determined in an iterative fashion without formally rearranging Equation 1. Bayesian statistical approaches are well suited to the problem of inverse prediction, but numerical calculations can be time consuming, and the development of a model requires familiarity with Bayesian statistics. Second, non parametric approaches are based directly on observed distributions of the data. These approaches are powerful because they do not require assumptions about error structure within the data to be made, but they also require that the data comprehensively represent the system. Third, the “fiducial” approach, which was never widely adopted as a broad statistical framework, and is all but absent from the modern statistical literature, is nevertheless widely used in the specific example of uncertainty estimation in inverse‐prediction problems (Williams, 1959). We refer the reader to (Draper & Smith, 1998) for a full description of this approach. Note that the “prediction interval” (PI), or root mean‐squared error (RMSE) of an “*E*‐on‐*P*” OLS regression (i.e., with *E* on the *y*‐axis and *P* on the *x*‐axis) is not a valid estimate of uncertainty in ${\hat{E}}_{0}$. This approach violates the assumptions of OLS regression, resulting in an incorrect slope and intercept, and an underestimate of the unexplained variance. Given the ubiquitous use of calibration‐based estimates throughout the geosciences, and the inconsistent calculation of uncertainty, there is a pressing need for an uncomplicated, robust and generalizable way to estimate the IPI.

### 3.2 Recommended Method for Simple Calculation of the IPI

We describe a simple and robust approach for estimating the IPI that can be straightforwardly applied to any calibration data set. In this approach we make the simplifying assumption that $\beta_{1}$ and $\beta_{0}$ are known with absolute certainty. Uncertainties in $\beta_{0}$ and $\beta_{1}$ are, of course, never zero, but this simplification is usually reasonable when the calibration data set shows a visually apparent correlation. If ϵ is an approximately normal distribution, it will be best represented for a finite sample size by a Student's *t*‐distribution. The value of $E_{0}$ corresponding to a single value of $P_{0}$, to within an uncertainty α is then:

(3) $$E_{0}={\hat{E}}_{0}\;\pm \;\frac{\hat{\sigma }t_{\alpha ,n-2}}{\left|{\hat{\beta }}_{1}\right|},$$

where ${\hat{E}}_{0}$ is the classical estimator of *E* (Equation 2) and $\hat{\sigma }$ is the observed standard deviation of the residuals from Equation 1. $t_{\alpha ,n-2}$ is the dimensionless critical value of the Student's *t*‐distribution, where n is the number of calibration data points and a linear model has n−2 degrees of freedom. α is the level of confidence desired (i.e., for 95% confidence bounds, α = (1–0.95)/2 = 0.025). For n→∞, $t_{\alpha ,n-2}$ approaches the critical value of the normal distribution ($t_{0.025,\infty }$ = 1.96). This estimate of uncertainty is shown in Figure 1 as “Simple IPI” and is graphically intuitive as rotating the standard deviation of the residuals from the *P* axis onto the *E* axis (Figure S1). Equation 3 is equivalent to the “quick‐and‐dirty” estimate of the uncertainty in $E_{0}$ given by Demidenko et al., which finds an estimate of σ for a Cauchy PDF in the limit of large n (Demidenko et al., 2013, Equation 16).

Some deductions can be made directly from Equation 3. The uncertainty in $E_{0}$ is proportional to σ, and is independent of the intercept, $\beta_{0}$, but decreases with the magnitude of the slope, $\left|\beta_{1}\right|$. This inverse dependence leads to the correct units for $\sigma /\beta_{1}$, and inversion becomes impossible when the slope approaches zero. Even when parameters of the model ($\beta_{0}$, $\beta_{1}$ and σ) are known exactly, uncertainty in $E_{0}$ based on a single value of $P_{0}$ is limited by the scatter of data about the regression line, and is not decreased by increasing the number of calibration data.

### 3.3 Comparison of the Simple IPI Estimate With Alternative Approaches

Using an artificial calibration data set, we compare the Simple IPI to estimates from the well‐established fiducial approach, a nonparametric approach, and a Bayesian approach. For the fiducial approach we used Equation 3.2.6 from Draper & Smith, 1998, which results in curved inverse prediction bands. For the nonparametric approach, we assumed a constant uncertainty across all *E*, and defined the IPI to lie within the 2.5th and 97.5th percentiles of the distribution of $E_{i}-{\hat{E}}_{i}$ (i = 1, …, *n*; see Text S1 for details). Bayesian calibration and prediction can be done: (a) in two steps (e.g., as in Tierney et al., 2019; Tierney & Tingley, 2014, 2018), whereby the posterior distributions of the parameters of the forward model are first established before they are used to generate posterior distributions of $E_{0}$; or (b) in a single step (e.g., as in Gelman et al., 2004, 2020) whereby the posterior distributions of the parameters of the forward model and of the value of $E_{0}$ are all generated within a single simulation. The Bayesian approach shown in Figure 1 is a two step approach (see Text S2 for details).

We furthermore evaluated all of these approaches against an “empirical” Monte Carlo (MC) simulation of the error distribution associated with inverse prediction. This involved repeatedly simulating the offset between a “known” value of *E*, and the value of *E* predicted from Equation 2 (see Text S3 and Figure S2 for details). The nonparametric approach produces a slightly asymmetric uncertainty range, and systematically underestimates the IPI at low n (Figure S3). The fiducial approach produces flared outward uncertainty range reflecting the effect of uncertainty in the slope, and systematically overestimates the IPI at low n (but is invalid at very low n; Figure S3). Estimates from the Bayesian and Simple IPI approaches lie closest to the simulated uncertainties. The Bayesian approach predicts slightly larger uncertainties at low n, and flares outwards very slightly due to uncertainties in the slope. All approaches converge on the same IPI as the MC simulation in the limit of large n (Figure S3). Further details of this simulation are given in the supporting information (Text S3). We also provide example *R* and *stan* (Stan Development Team, 2020) scripts for making these calculations (https://github.com/QGeoBio/IPI).

The calibration data set only needs to be large enough to define the three parameters of the model ($\beta_{0}$, $\beta_{1}$, and σ) with some confidence. Further increasing the size of the calibration data beyond this point does not significantly improve uncertainty in prediction (compare IPIs in Figures 1a and 1b and S3). For our artificial data sets, a “sufficiently large” data set to ignore parameter uncertainties is around 30–50 well‐spaced data points (Figure S3), but the controls on this value of n are a subject of future research. For a single proxy measurement, the IPI is determined by the amount of unexplained variance in the calibration data set (more scatter = greater uncertainty; compare Figures 1a and 1c), and the slope of the regression (shallower slope = greater uncertainty; compare Figures 1a and 1d). Uncertainties in model parameters (which do change with increased sample size; compare CBs in Figures 1a and 1b) contribute only a small fraction of the total prediction uncertainty. From these comparisons, for calibration data sets exhibiting a visually reasonable correlation between *E* and *P*, the Simple IPI approach (Equation 3) is an excellent estimate of uncertainty.

## 4 Example

**Figure 2:**
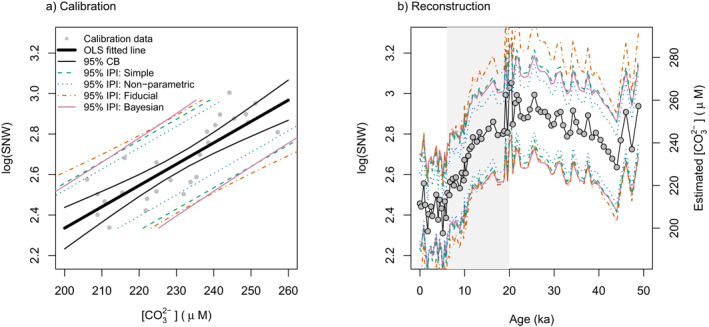
*Example:* Surface seawater carbonate ion concentration ([${CO}_{3}^{2-}$] in μM) reconstructed using size‐normalized foraminifera shell weights (SNW) over the last glacial cycle (Barker & Elderfield, 2002). The data shows a decrease in [${CO}_{3}^{2-}$] from the last glacial maximum (∼20 ka) to the mid‐Holocene (∼6 ka). Note that for the calibration we applied ordinary least squares (OLS) linear regression to the log transformed size‐normalized weight (SNW) data whereas Barker and Elderfield (2002) fit an exponential curve to the SNW data. Inverse prediction intervals calculated were based on the calibration data set presented in the same study. See text for discussion and Figure 1 for details of each approach.

Figure 2 shows a reconstruction of the surface ocean carbonate ion concentration ([${CO}_{3}^{2-}$]) across the termination of the last glacial cycle (Barker & Elderfield, 2002) using the foraminifera shell‐weight proxy. This proxy is based on the size‐normalized weight (SNW) of planktic foraminifera shells, which is shown to be higher at higher [${CO}_{3}^{2-}$]. Based on this simple univariate core‐top calibration, the 95% IPI is approximately ± 20 μM (Figure 2a). The authors concluded that reconstructed [${CO}_{3}^{2-}$] decreased from the last glacial maximum (∼20 ka) to the mid‐Holocene (∼6 ka) consistent with changes in carbonate chemistry expected from the timing and magnitude of ${CO}_{2}$ change observed in ice core records (Figure 2b). The inferred direction of the [${CO}_{3}^{2-}$] change is robust to the 95% level, but the magnitude of the change may be within a factor of 5 (max change = Δ[${CO}_{3}^{2-}$] = 110 μM from 290 to 180 μM; min change = Δ[${CO}_{3}^{2-}$] = 20 μM from 240 to 220 μM; see Figure S4).

If the following were true: (a) the nature of the samples in the calibration and the reconstruction were the same; (b) the signal was driven by [${CO}_{3}^{2-}$]; and (c) the scatter in the calibration and the time series was all noise (i.e., random), the noise in the time series of SNW of planktic foraminifera would reflect the noise in the calibration (i.e., ϵ). In this example, the coherency (autocorrelation) of the signal is very high, and the scatter in proxy data is relatively low. While this suggests that the signal in the proxy data is real (i.e., there was significant decrease in SNW of planktic foraminifera shells through this interval), there is a substantial uncertainty associated with how much of this signal was driven by [${CO}_{3}^{2-}$] (Figure 2b). The value of ϵ derived from the core‐top calibration is assumed to reflect natural variability, and its translation to an uncertainty in reconstructed [${CO}_{3}^{2-}$] implicitly includes the possibility that the observed trend in the SNW of planktic foraminifera shells could be driven by alternative additional influential variables (e.g., temperature [Qin et al., 2020] or nutrient concentration [Aldridge et al., 2012]).

## 5 Discussion

We restrict our discussion to the statistical perspective, with the following caveats: Calibrations, and the assumptions necessary for their application, must be representative throughout the geologic interval of interest. In other words, an assumption of uniformitarianism (sensu. Hutton [1785]) at the level of an observed relationship between *E* and *P* is the assumption that the model parameters, $\beta_{0}$, $\beta_{1}$ and σ, are constant through time. If the defined relationship is purely empirical, such that the mathematical form of the relationship is not mechanistically prescribed, and the coefficients of the relationship do not have a physical meaning, it is difficult to evaluate the validity of this assumption, or justify extrapolation of the model beyond the calibration data. The integrity of the measured characteristic of the material on which the proxy is based must also be unchanged from the state in which it appears in the calibration, to the state in which it is used for a reconstruction, and not be significantly impacted by intervening processes (e.g., transport or diagenesis).

### 5.1 Calibration Data: Experiments Versus Natural Samples

Calibrations based on experiments and calibrations based on natural samples provide different constraints on the model parameters. Constraining the coefficients of the linear model, $\beta_{0}$ and $\beta_{1}$, is usually the primary goal of regression analysis. For this, laboratory experiments are arguably the most reliable approach because *E* can be varied in isolation and both *E* and *P* can usually be measured with low uncertainty. By contrast, in natural samples, environmental variables are often not measured directly, but instead are taken from global or regional databases that are binned in space and time, which introduces uncertainty in the value of *E* that corresponds to the measured value of *P*. For *P*‐on‐*E* OLS regression, uncertainty in *E* has the potential to result in an underestimate of the slope (regression dilution), and may necessitate an errors‐in‐variables alternative to regression such as reduced major axis regression (Draper & Smith, 1998), York regression or Bayesian methods. While experiments can be used to constrain predictor coefficients, in the real world *P* depends on various quantities that span the predictor parameter space. What if *E*—chosen because it is the environmental variable of interest—is not the variable exerting the strongest control on *P* in nature? The choice of cross‐section through environmental parameter space represented by the experiments may lead to a significant interpretative bias during subsequent application if a calibration based on experiments is treated as directly representative of the natural world (e.g., Evans et al., 2018; Gray & Evans, 2019; Peterse et al., 2012).

Understanding the nature and magnitude of ϵ, meanwhile, is an often overlooked goal of regression analysis. Consider *P* as a function P (*E*, A, B) where A are stochastic variables that are responsible for some noise even under ideal controlled laboratory conditions and B are stochastic variables that represent environmental factors other than *E* or A. A might reflect instrument precision or biological variability, while B reflects any environmental variable that can be held constant under laboratory conditions, but may vary in the natural world. In a linear regression model between *P* and *E*, A and B are treated as a combined noise term, ϵ. Note that the Gauss‐Markov assumptions for OLS regression require that the mean of the noise in the dependent variable must be everywhere zero—thus, values of the environmental variables reflected by B must themselves be uncorrelated with E. Variances are usually additive, so one might expect that ϵ is larger in field samples compared to laboratory samples. Indeed, more unexplained variance in natural samples compared to laboratory experiments can be seen as an indication that another parameter not considered in laboratory studies is influential in the natural world. However, this is complicated by the potential for averaging (see below discussion).

Paleoenvironmental reconstructions always involve natural samples, so the value of ϵ used to calculate the IPI includes both *A* and *B*— this is information that can only be generated with a calibration based on natural samples, as a controlled laboratory experiment only provides information on *A*. In theory, it would be possible to comprehensively explore proxy parameter space with experiments. In practice, this is very difficult to do. The dimensionality of the relevant parameter space is often unknown before it is explored, and exploration is limited by the constraints of time and cost of experiments. “Double calibrations”, including both laboratory experiments and natural samples, may be a necessary compromise to isolating the effects of influential parameters, and estimating the fraction of real world variation that can be quantified.

### 5.2 Noise Versus Systematic Uncertainty and Replicate Analyses

Under certain circumstances, the uncertainty in $E_{0}$ can potentially be reduced by replicate measurements of $P_{0}$. However, whether this is possible depends on the nature of the unexplained variance in the calibration, and the nature of the noise in the paleoenvironmental samples. From the perspective of replication, the unexplained variance in the calibration can be thought of as a combination of: (a) noise, which is entirely stochastic, and which represents the range of measured values generated from repeated sampling of the “same” environment; and (b) systematic unexplained variance which is the result of variability in an influential environmental variable other than *E*. (a) would include all of the laboratory‐based stochastic variables referred to as A above, but might also include some variables referred to above as B, including any natural events that are stochastic such as weather. (b) might include the effects of diagenetic status, or any environmental parameter that varies in a non‐stochastic way in space and time, and between paleo‐samples. The component of uncertainty due to unexplained variance of type (a) is reduced with replicate sampling, whereas that due to type (b) is usually not.

In principle, if the unexplained variance in the calibration was all noise ([a] above), and if the nature of the calibration data and the paleo‐data were the same, the noise in a paleo‐time series in *P* should reflect the noise in the calibration. In this ideal case, true replicates at each time point would reduce the uncertainty in the reconstruction. However, it is usually not possible to distinguish between (a) and (b), and similarities between the magnitude of the noise in the calibration and the paleo‐data, are only circumstantial evidence of their common origin. Furthermore, there is ambiguity over the nature of a true replicate. Measurement replicates can account for instrumental error, and sample replicates can account for sediment heterogeneity, but are these true replicates of sampling the same environment? In reality, the definition of a true replicate depends on the proxy under consideration.

Further complications arise when the nature of the calibration samples and the target samples are different. For example, consider a hypothetical proxy based on material produced at the surface of a body of water. The calibration relies on sediment trap samples with seasonal changes in environment, while reconstructions are based on sediment samples. In this scenario, the analyzed material in both sediment trap samples and down core samples has been subjected to multiple influential environmental variables. However, each data point in the calibration represents a short interval of time (weeks to months), whereas a 1 cm depth interval of sediment would contain the integrated signal over years to tens of thousands of years, depending on the rate of sedimentation, and depth of bioturbation. If the reconstructed time series contains less noise than (unexplained variance in) the calibration, one possibility may be that the nature of the sediment samples smooths out noise or seasonal effects. Another explanation may be that the down‐core signal is skewed toward one season because a larger fraction of material produced at one time of year is transported to depth and archived in the sediment. If the reduced scatter is due to a reduction in noise through temporal averaging, this might be reproduced in the calibration—and thus accounted for—through replicates, based on separate but closely spaced sediment traps (targeting the “same” environment). In the case of seasonal skew, it would be necessary to develop a mathematical model to relate the sub‐annual surface signal to the export flux using additional information about the environment to determine the integrated contribution to the sediment throughout the year.

If a time series with substantially lower noise than the calibration is produced, either through the use of true paleo‐replicates, moving average (MA) or local regression (LOESS or LOWESS) approaches, or as a result of smoothing through the nature of sediment accumulation, there can be some confidence that any signal in the data represents a real change. However, this does not help to constrain the nature of the change. Unless the impact of influential variables other than *E* can be quantified or discounted, the uncertainty in the reconstruction of a change in *E* must be large enough to include the possibility that it is a non‐*E* environmental variable responsible for driving the observed change in *P*.

### 5.3 Relative Environmental Change

Often, the goal of paleoclimate reconstruction is to determine the magnitudes of relative change of an environment over a period of time. A common misconception arises when relative change at the same core location is assessed. Intuitively, trends or anomalies in the reconstructions based on data from samples from the same core location should be associated with far smaller uncertainties than values compared between different sites. In this case relative change is often reported with only analytical uncertainties, or the uncertainty in the slope. To understand whether this is appropriate, consider an end‐member scenario where the calibration data set consists of an infinite number of data points and there is zero uncertainty in the slope. It must be incorrect that zero uncertainty in the slope corresponds to zero uncertainty in the reconstructed value of *E*, because environments change in a multivariate fashion, and random events impact the value of *P*.

When the magnitude of the IPI is large relative to the magnitude of the signal in the proxy data, the reconstructed environmental change cannot be reported as robust. However, there is a subtle but fundamental distinction between asking (a) “what change in the environmental variable (ΔE *is* reflected by the observed change in *P* (Δ *P*)?”, compared with the much weaker question, (b) “what ΔE *could* explain the observed Δ *P*?”. The estimate is the same for both of these questions (calculated from Equation 2), but there is a large difference in the associated uncertainty. The uncertainty associated with (b) corresponds to the uncertainty in the slope of the calibration line ($\beta_{1}$), while that associated with (a) has an uncertainty corresponding to the (much larger) IPI. This difference in uncertainties reflects the strength of the statement. When assessing the plausibility of a hypothesis, it may be appropriate to quote the uncertainty associated with (b). However, comparisons between different proxies, or between proxies and models, always requires that the uncertainty associated with (a) is used. We suggest that this distinction is made explicit whenever paleoclimate data are presented.

### 5.4 Influence of Multiple Environmental Factors

Systematic uncertainty can be accounted for through the inclusion of additional explanatory variables in to a multiple regression model. This requires higher dimensional data sets and more complex models (e.g., Evans et al., 2016; Peterse et al., 2012; Tierney & Tingley, 2014). Note that the accurate attribution of the effect of each environmental variable on the value of *P* is limited if the explanatory variables are correlated (exhibit collinearity or temporal auto‐correlation) but cannot be assumed to perfectly co‐vary on all relevant spatial and temporal scales (Kutner et al., 2004). This is extremely common in natural environments. When multiple regression models (based on uncorrelated predictors) are used for reconstructions, explicit assumptions can be made about the likely ranges of these additional variables to constrain the parameter space.

A promising future direction to this approach could include the combination of multiple measurements with common explanatory variables into a multiple multivariate analysis. If k measurable characteristics of the same system ($P^{\left(1\right)}$, $P^{\left(2\right)}$ … $P^{\left(k\right)}$) are all functions of an equal number of environmental parameters ($E^{\left(1\right)}$, $E^{\left(2\right)}$ … $E^{\left(k\right)}$), a model relating the control of $E^{\left(1:k\right)}$ to $P^{\left(1:k\right)}$ could be inverted to constrain $E^{\left(1:k\right)}$ from measurements of $P^{\left(1:k\right)}$ simultaneously. When multivariate data are considered together, there is potential to place tighter constraints on the system as a whole than is possible with a single explanatory variable alone.

## 6 Summary and Outlook

In summary, any variance in the calibration that cannot be explained must be reflected in the uncertainty in the reconstruction. We provide a simple method to calculate this uncertainty (Equation 3). The focus must, therefore, be on accounting for as much of the variance in the calibration data as possible, either through appropriate replicate measurements, through additional explanatory variables, or through an explicit mechanism relating calibration and down‐core data. The uncertainty associated with inverse prediction for a calibration with a single explanatory variable is not significantly improved with the addition of more data once the parameters of the model ($\beta_{0}$, $\beta_{1}$ and σ) are reasonably well constrained. The controls on what constitutes a sufficiently large data set to ignore parameter uncertainties is a subject of future research. We, therefore, suggest that the emphasis should shift away from the generation of calibration data sets consisting of a very large number of data points comprising a single explanatory variable, and toward data sets that consist of fewer data points that comprise multiple explanatory variables.

In an ideal world, one would have the same level of noise in laboratory experiments, modern field data and paleo data. However, one would expect that the noise level increases from laboratory experiments to modern field data and further to paleo data because of additional influences of processes not represented in the laboratory or on short time scales. The differences in noise levels could provide clues about the nature of deviations from the ideal world. In reality, the noise level of paleo data might be even lower than those of the corresponding lab data because of smoothing due to averaging.

Paleoclimate proxies usually have calibrations that contain a relatively large amount of unexplained variance, which translates to large uncertainties in reconstructed values. We advocate for an approach where the measurement data are presented as the direct observations, and the environmental reconstruction can, subsequently, be presented as a plausible interpretation. The uncertainty in the magnitude of environmental change that would *plausibly* explain the observation, corresponds to the uncertainty in the gradient ($\beta_{1}$). Absolute reconstructions must carry the full associated uncertainty of the IPI. This distinction between assessing the plausibility of a hypothesis, and reconstructing absolute changes or states of environment, is subtle but of fundamental importance when comparing proxy records or integrating proxies with models.

## Supporting information

Supporting Information S1

## Data Availability

The data for the example in Figure 2 are available from Barker and Elderfield (2002); data were otherwise not used nor created for this research. *R* and *Stan* scripts for the calculations described in this manuscript are available on GitHub (https://github.com/QGeoBio/IPI), and an interactive web application is under development and will be available at: http://www.qgeobio.com/IPI.
