# Supplementary material for: Statistical Uncertainty in Paleoclimate Proxy Reconstructions
Source: Geophys Res Lett. 2021 Aug 1;48(15):e2021GL092773. doi: 10.1029/2021GL092773 (PMC9285564; doi:10.1029/2021GL092773)
Supplement: Supplementary file 1 — Supporting Information S1 [file GRL-48-0-s001.pdf]

Supporting Information for "Statistical uncertainty in paleoclimate proxy reconstructions"

H. L. O. McClelland[1,2,3], I. Halevy[3], D. A. Wolf-Gladrow[4], D. Evans[5], A. S. Bradley[2]

[1]School of Earth Sciences, University of Melbourne, Australia

[2]Department of Earth & Planetary Sciences, Washington University in St. Louis, St Louis, USA

[3]Department of Earth & Planetary Sciences, Weizmann Institute of Science, Rehovot, Israel

[4]Alfred Wegener Institute for Polar and Marine Research, Bremerhaven, Germany

[5]Institute of Geoscience, Goethe University Frankfurt, Frankfurt, Germany

Contents of this file

1. Text S1 to S3

2. Figures S1 to S4

Introduction

This supplementary information provides additional methodological details and figures.

Text S1. A non-parametric estimate of the IPI

A non-parametric measure of the uncertainty in the estimate of E is obtained through direct comparison of modeled estimates of E with observations. Values of $\hat{E}_i$ are generated by passing the calibration values of P_i to Eq. 2. A non-parametric estimate of the IPI can be made by directly determining the desired quantiles of the distribution of differences between all of the observed E_i and model-predicted $\hat{E}_i$ (i.e., the 2.5^{th}, and 97.5^{th} percentiles, for 95% confidence level). This estimate of uncertainty is shown as the non-parametric estimate of the IPI in the manuscript. Non-parametric approaches are powerful – they require few assumptions, and can be asymmetric about the regression line, but rely entirely on the raw data and do not extrapolate beyond it. This approach, therefore, works best with large calibration data sets.

Text S2. A Bayesian estimate of the IPI

The Bayesian estimate of the IPI shown in Figs 1, 2, S2 and S3 is calculated in two steps. The first step is calibration, where calibration data is used to generate posterior probability distributions for β_0, β_1, and σ. The linear relationship between P and E for the calibration data set can be re-written:

$$P_i \sim \mathcal{N}(\beta_0 + \beta_1 E_i, \sigma^2). \tag{1}$$

The second step is prediction, where the posterior probability distribution for E_0 is generated. The relationship between a new value of P, P_0, and the corresponding value of E, E_0, is given by:

$$P_0 \sim \mathcal{N}(\beta_0 + \beta_1 E_0, \sigma^2), \tag{2}$$

where β_0, β_1 and σ are posterior probability distributions calculated in Eq.1. A posterior distribution in E_0 is generated for each realized calibration parameter set, and all values are combined for a total posterior distribution in E_0. We used flat priors for the slope and intercept parameters, and for the values of E_0. The prior for σ was flat, but strictly positive. All calculations were done numerically with the R interface to the software Stan (code available at: https://github.com/qgeobio/IPI).

Text S3. Monte Carlo simulation

The reliability of the methods used to estimate the IPI were evaluated with a Monte Carlo simulation (Figs S2 and S3). The details of this approach are as follows (also depicted in Fig. S2.A): First, as for the analysis shown in Fig. 1, an artificial calibration data set of n pairs of E and P values was generated by choosing 'true' values of the intercept and slope (β_0 and β_1), and σ (with ϵ assumed normal), and passing randomly chosen E values to Eq. 1 to generate corresponding P values (i.e. (E_i, P_i), i = 1, ..., n). An OLS linear regression model was then fitted to the artificial dataset to generate estimates of β_0 and β_1 ($\hat{\beta}_0$ and $\hat{\beta}_1$ respectively). The Monte Carlo simulation was then initiated by inserting one further 'true' E value (E_0) into Eq. 1 to generate a corresponding P value. This P value was then treated as a new measurement (P_0) and inserted to Eq. 2, using the fitted estimates $\hat{\beta}_0$ and $\hat{\beta}_1$, to generate a value of the classical estimator for E ($\hat{E}_0$) for the new P_0. As the 'true' value of E, E_0, is known, the error in the estimate, $\hat{E}_0$, can be calculated (i.e. $\Delta E = \hat{E}_0$ - E_0). This was repeated a large number ($\sim 10^6$) of times. The values of ΔE corresponding to a narrow band around a specific P_0 value were taken, and the 2.5th

and 97.5th percentiles of this distribution are presented as the Monte Carlo simulation or the error in E for that value of P (Fig. S2.B and S2.C). This analysis simulates an 'empirical' distribution of estimates of E for a given artificial calibration data set.

This entire approach was repeated with randomly generated artificial calibration data sets of different sizes to evaluate the impact of n on the reliability of statistical estimates of the IPI (Fig. S3). 100,000 artificial datasets were generated ranging from $n = 3$ to $n = 1000$. The MC simulation is considered as an empirical representation of the error distribution associated with estimates of E_0 for a given calibration dataset. At high n the estimate of the IPI is not reduced with the addition of data points. At very low n ($n < 8$), the calibrations are not reliable, and the fiducial approach is unstable in this region. At low to medium n ($8 < n < 30 - 50$), the non-parametric approach systematically underestimates the IPI while the fiducual approach systematically overestimates the IPI. The Bayesian approach and the Simple IPI approach described in the main text are a good estimates of the IPI. At high n ($n > 30 - 50$) all approaches converge to the same value of the IPI. As can be seen here, there are substantial increases in the reliability of calibrations with increasing n, up to around $n = 30 - 50$. Beyond this, increasing n results in only modest improvements.

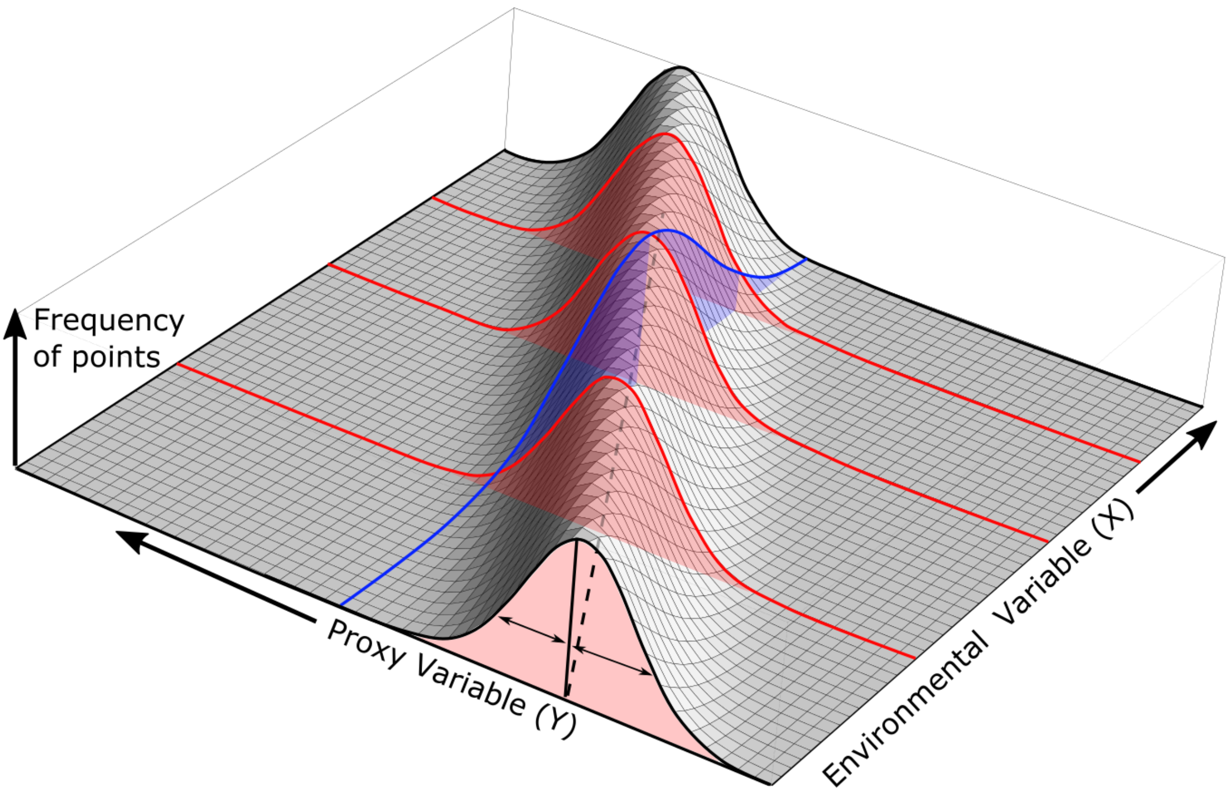

Figure S1. Graphical representation of the Simple IPI. The surface in this illustration represents the probability distribution of the response variable (P: the proxy variable, plotted on the y axis) across a continuous range of values of the independent variable (E: the environmental variable, plotted on the x axis). The red lines and corresponding shaded regions represent the probability distribution at 3 different values of the environmental variable (noise in the regression). The blue line and corresponding shaded area represent the translation of the probability distribution in the y axis to the x axis by 'rotating' the distribution through the gradient. This represents the 'Simple IPI' approach outlined in the main text (Eq.3).

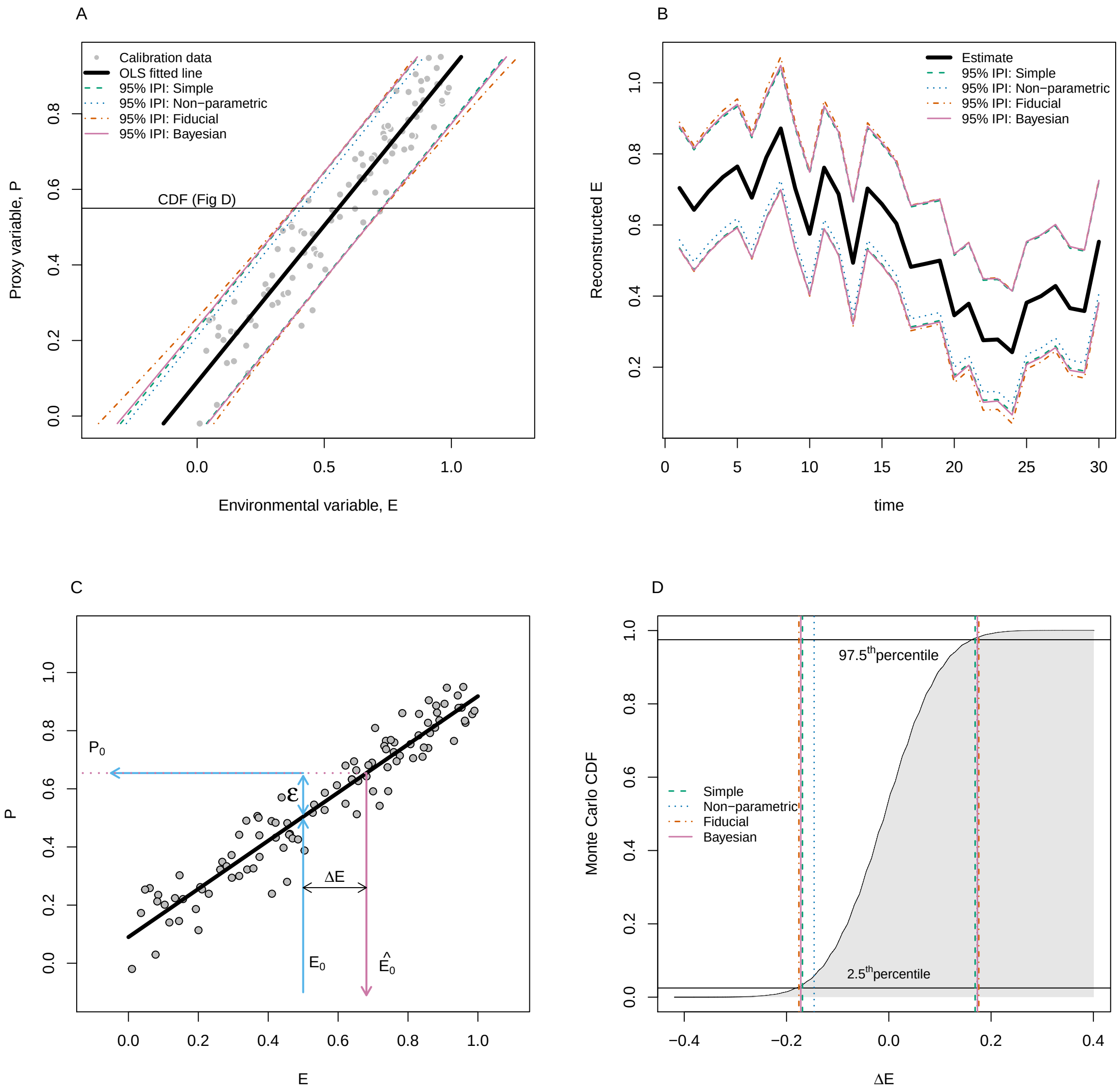

Figure S2. Validation of IPI estimation methods. A) Example calibration based on synthetic data. **B)** Example application to synthetic time series data. **C)** Illustration of the Monte Carlo (MC) simulation and definition of ΔE (see Text S3 for description of the approach). The value ΔE ($\hat{E}_0$ - E_0) represents the offset of the estimate of E from the 'true' value of E. **D)** Cumulative distribution function (CDF) of ΔE from the Monte Carlo analysis at a given P value (labeled in A), with IPI estimates plotted for comparison. Parameters: $\beta_0 = 0.1$; $\beta_1 = 0.8$; $\sigma = 0.07$; $n = 100$. Code available at: https://github.com/qgeobio/IPI

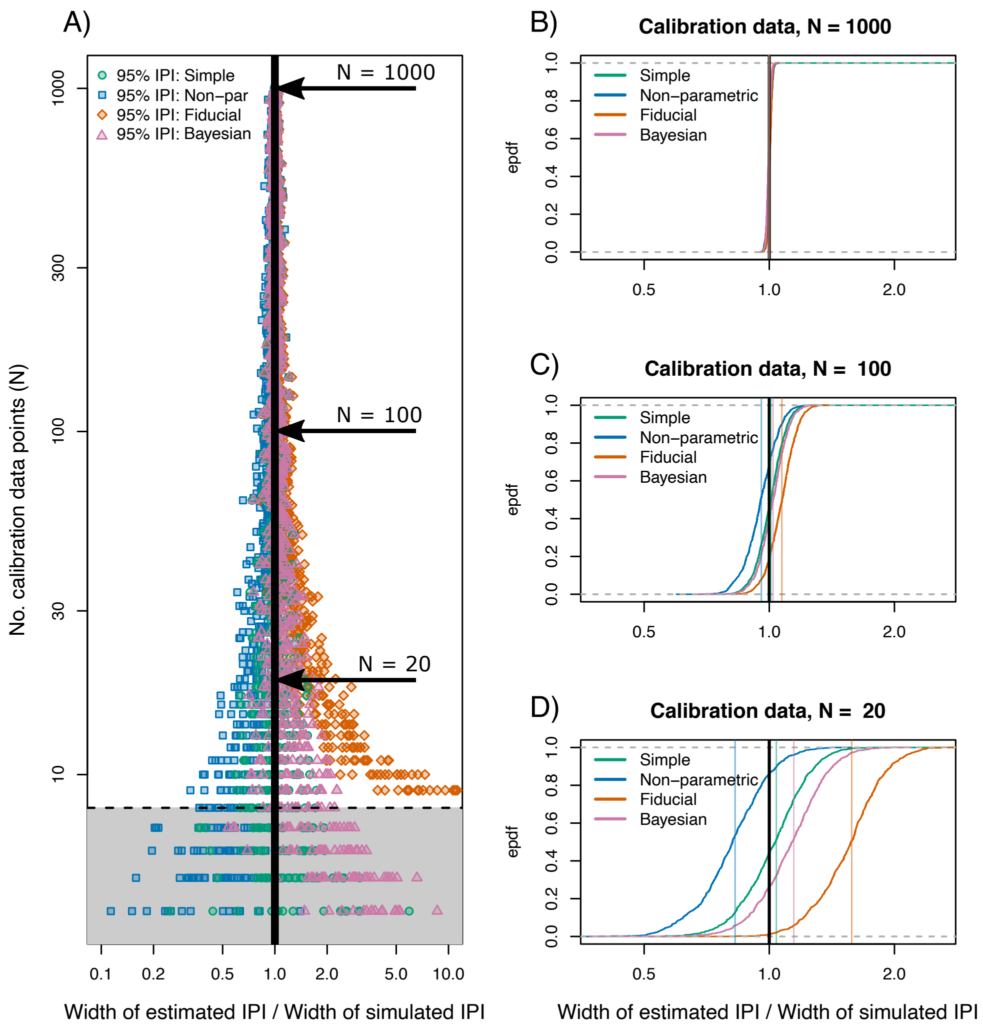

Figure S3. Effect of n on IPI for different methods. A) Estimates of the width of the IPI, calculated using four different methods, at a wide range of differently-sized calibration data sets (no. data points = n), relative to the width of the IPI from the MC simulation. **B)** Empirical cumulative distribution function (ecdf) of the width of the estimated IPI relative to the MC IPI for four different methods, for a calibration dataset consisting of $n = 1000$ datapoints. Each ecdf is formed of 5000 MC simulations. **C)** As B but with $n = 100$. **D)** As B but with $n = 20$. The parameters of the artificial calibration data sets are: $\beta_0 = 0.3$; $\beta_1 = 0.5$; $\sigma = 0.05$; and variable n.

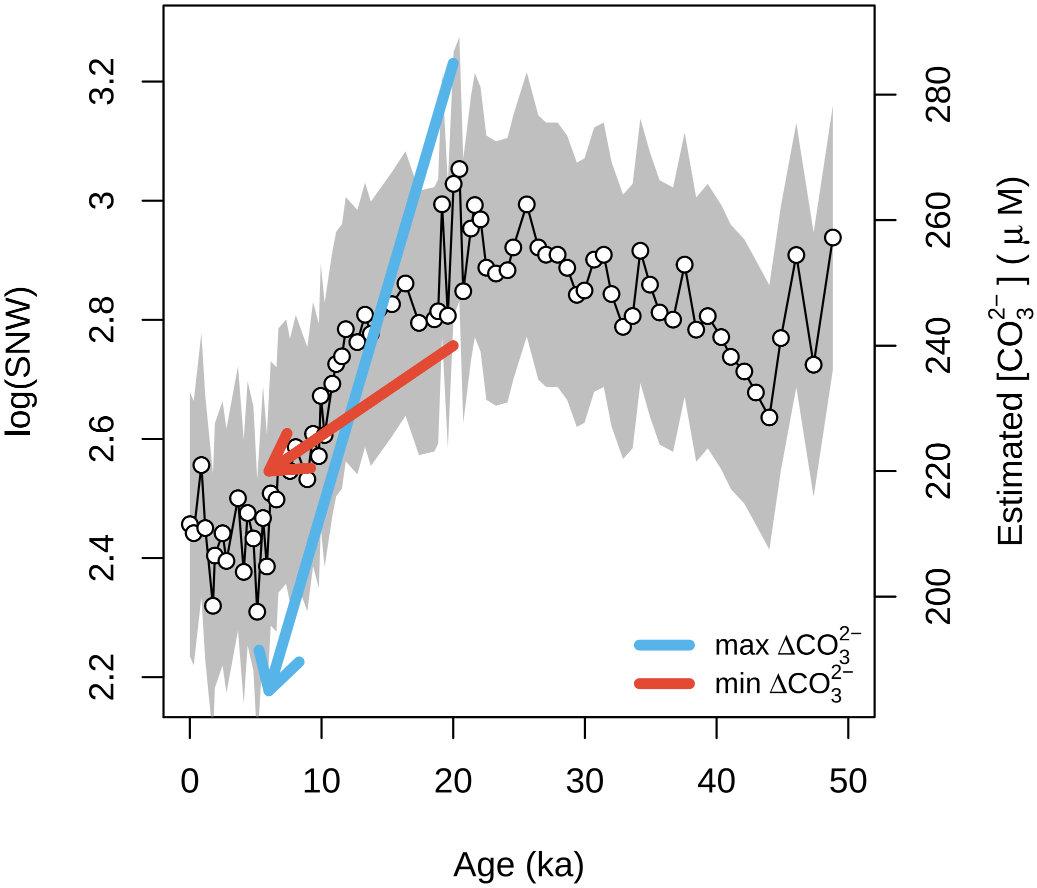

Figure S4. Maximum and minimum inferred paleoenvironmental changes for a given proxy time series. Annotated version of Figure 2B from the main manuscript. Grey region represents the 'simple IPI'. Red and blue arrows respectively depict the minimum and maximum inferred change in $[CO_3^{2-}]$ for the observed change in SNW of foraminifera shells – i.e. the minimum and maximum change possible within the shaded region.
